# Mono- versus polydrug abuse patterns among publicly funded clients

**DOI:** 10.1186/1747-597X-2-33

**Published:** 2007-11-08

**Authors:** Satish Kedia, Marie A Sell, George Relyea

**Affiliations:** 1 Institute for Substance Abuse Treatment Evaluation (I-SATE), The University of Memphis, 316 Manning Hall, Memphis, Tennessee, 38152, USA; 2 Center for Community Health, The University of Memphis, Billy Mac Jones Hall, 633 Normal Street, Memphis, Tennessee, 38152, USA

## Abstract

To examine patterns of mono- versus polydrug abuse, data were obtained from intake records of 69,891 admissions to publicly funded treatment programs in Tennessee between 1998 and 2004. While descriptive statistics were employed to report frequency and patterns of mono- and polydrug abuse by demographic variables and by study years, bivariate logistic regression was applied to assess the probability of being a mono- or polydrug abuser for a number of demographic variables. The researchers found that during the study period 51.3% of admissions reported monodrug abuse and 48.7% reported polydrug abuse. Alcohol, cocaine, and marijuana were the most commonly abused substances, both alone and in combination. Odds ratio favored polydrug abuse for all but one drug category–other drugs. Gender did not affect drug abuse patterns; however, admissions for African Americans and those living in urban areas exhibited higher probabilities of polydrug abuse. Age group also appeared to affect drug abuse patterns, with higher odds of monodrug abuse among minors and adults over 45 years old. The discernable prevalence of polydrug abuse suggests a need for developing effective prevention strategies and treatment plans specific to polydrug abuse.

## Background

Alcohol and illicit drug abuse continues to be a major public health concern in the United States. According to the U.S. Department of Health and Human Services' (DHHS) 2005 National Survey on Drug Use and Health, 6.6% (16 million) of Americans age 12 or older reported heavy drinking, 22.7% (55 million) reported binge drinking, and 8.1% (19.7 million) reported using illicit drugs within the month prior to the survey [1]. A DHHS survey in 2002 found that 56% of all admissions to publicly funded treatment facilities were for multiple substances; among these admissions, 76% abused alcohol, 55% abused marijuana, 48% abused cocaine, 27% abused opiates, and 26% abused other drugs [2]. Despite the high rates of polydrug abuse, the literature on this issue is limited. In addition, there are few studies reporting the probability of mono- versus polydrug abuse across various demographic characteristics. Drawing upon 7 years (1998–2004) of admission data, this study examines the patterns of mono- versus polydrug abuse among admissions to publicly funded alcohol and drug abuse treatment programs in Tennessee.

The World Health Organization defines polydrug abuse as the concurrent (taken at the same time) or sequential (one drug taken followed by another) abuse of more than one drug or type of drug, with dependence upon at least one [3]. Such abuse has been increasingly reported in emergency room admissions and has been linked to drug-related deaths as well as nonfatal overdoses [4-7]. The abuse of multiple substances has a long recorded history in the United States, dating to the early 20^th ^century when combination drug abuse was found among 11.9% of narcotic addicts in New York and 1% of patients in Louisiana [8]. During the 1960s, polydrug abuse increased drastically; for example, barbiturate abuse was noted among 29% of heroin addicts and 32.4% of narcotic addicts in Lexington, Kentucky, as well as among the urban heroin addicts who would alternate alcohol with illicit drug abuse, at times as a detoxification method [8].

Alcohol abusers often report abuse of other substances. In a study of 248 alcoholics seeking substance abuse treatment, 68% reported using additional drugs in the 90 days prior to admission [9]. Another recent study found that over 80% of the alcoholics in treatment were dependent on at least one other substance, most often marijuana or cocaine. Conversely, alcohol abuse was noted among admissions who were primarily in treatment for dependence on illicit drugs–84% for cocaine, 71% for barbiturates, 67% for opiates, and 64% for hallucinogens [10].

Other studies have revealed a prevalence for polydrug abuse as well. Darke and Hall found that monodrug abuse was rare among 329 heroin abusers and 301 amphetamine abusers during a 6-month study period [11]. Leri et al. surveyed 1,111 injection drug abusers regarding their combination of cocaine and heroin abuse and found that approximately 15% not being treated in methadone maintenance programs were co-abusers of the drugs. This study also found that the individuals who abused both drugs tended to abuse them sequentially as opposed to simultaneously [12]. Additionally, researchers have noted that polydrug abusers who combine cocaine and heroin do so to achieve specific effects not attainable with just one substance; this practice can transform primary abuse of one drug into a pattern of polydrug abuse [13].

While there has been some research examining predictive variables associated with polydrug abuse, these studies have focused on psychological aspects of the abusers, including high scores on various psychometric scales [14] or a range of psychosocial factors such as depression, psychological distress, family support/bonding, and social conformity as a proxy for traditional values [15]. The study presented here statistically examines demographic characteristics and drug abuse patterns of a large statewide dataset of publicly funded admissions to substance abuse treatment programs over a 7-year period (1998–2004) in Tennessee. The goals of this paper include: 1) determining patterns of mono- or polydrug abuse; 2) identifying the most prevalent combinations of polydrug abuse; and 3) examining the probability of mono- versus polydrug abuse by demographic variables.

## Methods

### Data collection

Data used in this study were collected by the Division of Alcohol and Drug Abuse Services (hereafter, the Division) of the Tennessee Department of Mental Health and Developmental Disabilities from treatment agencies that are funded by DHHS' Substance Abuse Prevention and Treatment (SAPT) Block Grant, which provides over 75% of the publicly funded alcohol and drug abuse services in the state [16]. All treatment facilities receiving SAPT funds are required to collect data for each admission on the client being treated, including his or her demographics, substance abuse history and current patterns of abuse, and the criminal justice system involvement.

Because the data is collected for each admission, there can be multiple admissions representing the same client. A client who is admitted first to an inpatient residential facility and then is transferred to a halfway house will have two admissions in the same dataset. Thus, the number of treatment episodes is most likely overestimated. However, this approach is the same as that used by the federal Substance Abuse and Mental Health Services Administration (SAMHSA), which reports the Treatment Episode Data Sets (TEDS) based on all admissions (SAMHSA TEDS 1995–2005) for the SAPT Block Grant clients. Similarly, the dataset for this study is limited to those clients who obtained publicly funded treatment. There was no mechanism in place for obtaining information about clients receiving treatment not funded by public funds. In addition, because the state of Tennessee neither collects data on nicotine use nor provides smoking cessation interventions through the Division, this study does not include nicotine in the examination of polydrug abuse as reported by a few other researchers [15,17-20].

Data in Tennessee are compiled into a statewide database using Insight-CH software, which has specific modules for demographic variables, such as gender, age, ethnicity, employment, occupation, marital status, household income, and living arrangement, as well as referrals to treatment, substance abuse history and patterns, frequency of abuse, routes of administration, arrest record and legal status, medical history and conditions, and the recommended American Society of Addiction Medicine (ASAM) level of treatment. This data is made available to the Institute for Substance Abuse Treatment Evaluation (I-SATE) at The University of Memphis for use primarily as baseline data for conducting an annual outcomes evaluation, but it is also available for further analysis and research. The University of Memphis's use of the data for both evaluation and research purposes is approved and monitored by the University's Institutional Review Board (IRB).

### Data analysis

Microsoft Access and Visual Basic were employed to prepare intake data, and SAS/Base and SAS/STAT were used for statistical analyses. Data were first analyzed to provide information regarding the study sample, including demographic characteristics and types of substances reported at admission. Substances were categorized as alcohol (beer, wine, liquor), cocaine (crack, coke, dust), marijuana (hashish, pot, dope), opiates (heroin, methadone, OxyContin), sedatives (methaqualone, phenobarbital, Doriden), stimulants (amphetamine, methamphetamine, methylphenidate [Ritalin], MDMA [Ecstasy]), hallucinogens (LSD, peyote, mushrooms), inhalants (aerosols, solvents, nitrites, anesthetics, and others), and other drugs (mostly club drugs such as GHB/GBL, Ketamine, diphenhydramine, diphenylhydantoin/phenytoin, Dilantin). Descriptive statistics were used to report the frequency and patterns of mono- versus polydrug abuse across demographic variables and study years. Further analyses were conducted to assess the probability of mono- versus polydrug abuse for each substance category and to find the most frequent combinations of substances among polydrug abusers. In addition, bivariate logistic regression was applied to determine the odds of mono- versus polydrug abuse for each of the demographic variables and study years examined.

## Results

### Study participants

Over the 7-year period, there were 70,427 admissions to 183 agencies in Tennessee for treatment funded by the SAPT Block Grant. Those with missing drug abuse information (*n *= 536) were removed from the analysis, leaving a total of 69,891 admissions in the study sample. Of the admissions included, 68.9% were male and 90.0% were adults (18 years or older), with most (*n *= 42,564; 60.9%) falling between the ages of 25 and 44 (see Table 1). The majority were Caucasian (*n *= 46,910; 69.2%), one third were African American (*n *= 20,393; 30.1%), and only a few were of Hispanic or other ethnic origins (*n *= 495; 0.7%). The study sample was almost evenly divided between those living in urban (*n *= 35,062; 50.2%) and rural (*n *= 34,829; 49.8%) areas. Over the study period (1998–2004), the annual number of admissions to treatment programs in this study ranged from a low of 9,123 (13.1%) in 1999 to a high of 10,738 (15.4%) in 2003.

**Table 1:** Demographics of Study Sample (*N *= 69,891)

	Frequency	
	
Variable	*n*	%
Gender		
Male	48,168	68.9
Female	21,723	31.1
Ethnicity^*a*^		
Caucasian	46,910	69.2
African American	20,393	30.1
Other	495	0.7
Age^*b*^		
<18	6,955	10.0
18–24	10,701	15.3
25–34	20,671	29.6
35–44	21,893	31.3
45–54	8,266	11.8
≥ 55	1,404	2.0
Residential Area		
Urban	35,062	50.2
Rural	34,829	49.8
Year Admitted to Treatment		
1998	9,911	14.2
1999	9,123	13.1
2000	9,598	13.7
2001	10,214	14.6
2002	9,891	14.2
2003	10,738	15.4
2004	10,416	14.9

^*a*^2,093 missing ethnicity responses were removed from the total *N *for ethnicity percentages.

^*b*^1 missing age response.

### Patterns of mono- vs. polydrug abuse

Table 2 presents the drugs abused by the study sample, breaks the totals for each category into mono- or polydrug abuse, and reports the likelihood of mono- versus polydrug abuse for each substance. Alcohol was the most frequently reported substance (*n *= 45,318; 64.8%), followed by cocaine (*n *= 28,230; 40.4%) and marijuana (*n *= 24,671; 35.3%). Opiate abuse was reported by 12.4% (*n *= 8,652) of all admissions, sedatives by 6.7%, and other drugs by 6.0%. The least reported categories were amphetamines (*n *= 3,711; 5.3%), hallucinogens (*n *= 711; 1.0%), and inhalants (*n *= 467; 0.7%). Overall, slightly more admissions reported monodrug abuse (*n *= 35,875; 51.3%) than polydrug abuse (*n *= 34,016; 48.7%). Within each drug category, admissions were separated into mono- versus polydrug abusers. Of all admissions who reported alcohol abuse, 39.9% (*n *= 18,078) reported solely abusing alcohol compared to 60.1% (*n *= 27,240) who reported abusing alcohol along with some other substance. Similarly, 77.8% (*n *= 21,970) of the admissions reporting cocaine abuse and 79.9% (*n *= 19,710) of those using marijuana were polydrug abusers. Higher proportions of polydrug abuse were seen among the lesser abused drugs; over 90% of sedative and hallucinogen abusers and about 80% of amphetamine and inhalant abusers reported polydrug abuse. The category of "other drugs," mostly comprising club drugs, constituted the only category that had more mono- (*n *= 2,836; 68.0%) than polydrug (*n *= 1,336; 32.0%) abuse.

**Table 2:** Mono- vs. Polydrug Abuse Reported (*N *= 69,891)

	Total Users^*a*^		Monodrug Users		Polydrug Users			
	N = 69,891		35,875	51.3%	34,016	48.7%	Odds Ratio	
				
Substance	*n*	%	*n*	%	*n*	%	OR^*b*^	95% CI
Alcohol	45,318	64.8	18,078	39.9	27,240	60.1	0.664	0.651–0.670
Cocaine	28,230	40.4	6,260	22.2	21,970	77.8	0.285	0.277–0.289
Marijuana	24,671	35.3	4,961	20.1	19,710	79.9	0.252	0.244–0.256
Opiates	8,652	12.4	2,492	28.8	6,160	71.2	0.405	0.386–0.415
Sedatives	4,690	6.7	363	7.7	4,327	92.3	0.084	0.075–0.089
Amphetamines	3,711	5.3	752	20.3	2,959	79.7	0.254	0.235–0.265
Hallucinogens	711	1.0	42	5.9	669	94.1	0.063	0.046–0.074
Inhalants	467	0.7	91	19.5	376	80.5	0.242	0.192–0.273
Other drugs^*c*^	4,172	6.0	2,836	68.0	1,336	32.0	2.122	1.989–2.199

*Note: *Percentages for mono and polydrug abuse are calculated from Total Users of each substance across rows and not down columns; n values are actual number of users.

^*a *^This column reflects mono- and polydrug use for each particular substance category. Frequencies (*N*, %) do not add up to total users or 100% because of multiple responses among categories.

^*b *^For each drug, odds ratio indicates the likelihood of the client's being a mono- or polydrug user when reporting that substance as the one requiring treatment. Odds ratios were estimated from bivariate logistic regression analysis; all were significant at *p *< 0.0001.

^*c *^*Other *drugs refers mostly to club drugs, such as ecstasy, GHB, Ketamine, and Rohypnol.

Using bivariate logistic regression, an odds ratio for each drug was calculated to estimate the likelihood of mono- versus polydrug abuse. "Other drugs" showed more than twice the likelihood that a client would be a monodrug abuser (OR = 2.122, CI = 1.989–2.199). Individuals in the rest of the drug categories were significantly more likely to be polydrug abusers (ORs < 1.00, *p*-values < 0.0001). Despite only a marginal difference among all abusers (less than 3%) in the frequency of those reporting mono- versus polydrug abuse, analyses of individual drug categories explicitly showed a higher probability of polydrug abuse in all but one category.

### Prevalent combinations of substance abuse

Most polydrug abusers reported combining two or three substances (see Table 3). Over the 7-year period, roughly 30% (*n *= 20,789) of the entire client base reported using two substances, less than a fifth (*n *= 10,711; 15.3%) reported using three substances, and approximately 4% abused four or more drugs. While those who abused two types of substances reported 35 of the 36 possible combinations of two drugs, only 67 of the 84 possible combinations of three substances were reported, and even fewer of the possibilities combining four or more substances were reported by abusers. Substances abused in the most prevalent dyads or triads are presented in Table 4. The most common two-substance combination recorded was alcohol/cocaine at 12.0% (*n *= 8,374) of all admissions, followed by alcohol/marijuana (*n *= 5,182; 7.4%), cocaine/marijuana (*n *= 2,371; 3.4%), and alcohol/opiates (*n *= 916; 1.3%). Among three-substance abusers, the triad of alcohol/cocaine/marijuana was the most reported, abused by nearly 9% of the study sample (*n *= 6,202), distantly followed by alcohol/cocaine/opiates (*n *= 663; 1.0%). Each of the remaining two- and three-substance combinations accounted for less than 1% of all admissions.

**Table 3:** Combinations of Substances Used (*N *= 69,891)

Number of Substances Used	Possible Number of Drug Combinations	Number of Drug Combinations Found	Number of Admissions	
			
			*n*	%
1	N/A	N/A	35,875	51.3
2	36	35	20,789	29.8
3	84	67	10,711	15.3
4	126	68	1,833	2.6
5	126	46	478	0.7
6	84	29	140	0.2
7	36	13	47	0.067
8	9	6	17	0.024
9	1	1	1	0.0014

**Table 4:** Most Common Substance Combinations (*N *= 69,891)

Substance Combination	*n*	%^*a*^
Two substances (*n *= 20,789; 29.8%)		
Alcohol, Cocaine	8,374	12.0
Alcohol, Marijuana	5,182	7.4
Cocaine, Marijuana	2,371	3.4
Alcohol, Opiates	916	1.3
Cocaine, Opiates	593	0.9
Amphetamines, Marijuana	449	0.6
Opiates, Sedatives	445	0.6
Alcohol, Sedatives	429	0.6
Marijuana, Opiates	395	0.6
Alcohol, Other^*b*^	282	0.4
Alcohol, Amphetamines	260	0.4
Marijuana, Sedatives	187	0.3
Cocaine, Amphetamines	159	0.2
Marijuana, Other^*b*^	104	0.2
Combinations with *n *< 100	643	0.9
Three substances (*n *= 10,711; 15.3%)		
Alcohol, Cocaine, Marijuana	6,202	8.9
Alcohol, Cocaine, Opiates	663	1.0
Alcohol, Marijuana, Sedatives	483	0.7
Alcohol, Sedatives, Other^*b*^	430	0.6
Alcohol, Marijuana, Amphetamines	388	0.6
Alcohol, Opiates, Sedatives	327	0.5
Alcohol, Cocaine, Sedatives	275	0.4
Cocaine, Marijuana, Opiates	241	0.3
Alcohol, Cocaine, Amphetamines	167	0.2
Marijuana, Opiates, Sedatives	153	0.2
Cocaine, Marijuana, Amphetamines	151	0.2
Cocaine, Marijuana, Sedatives	129	0.2
Alcohol, Marijuana, Other^*b*^	125	0.2
Alcohol, Cocaine, Other^*b*^	118	0.17
Cocaine, Opiates, Sedatives	118	0.17
Combinations with *n *< 100	741	1.1

^*a*^Percentages are based on the entire client base and not just those abusing two or three substances.

^*b*^*Other *refers mostly to club drugs, such as ecstasy, GHB, Ketamine, and Rohypnol.

### Mono- versus polydrug abuse by demographic variables

Mono- and polydrug abuse examined by demographic variables using bivariate logistic regression yielded several notable results (see Table 5). For both male and female admissions, there was no difference in the likelihood of being a mono- or polydrug abuser (OR = 0.979, *p *= 0.130 and OR = 1.021, *p *< 0.10). Caucasian admissions were found to be twice as likely to be monodrug abusers (OR = 1.957, *p *< 0.0001) compared to other ethnicities, while African Americans were twice as likely to be polydrug abusers (OR = 0.504, *p *< 0.0001). Admissions of all other ethnicities, primarily Hispanics, were equally as likely to be mono- as polydrug abusers (OR = 1.154, *p *= 0.113).

**Table 5:** Likelihood of Mono- versus Polydrug Abuse by Demographic Variables and Study Years (*N *= 69,891)

	Total Users		Monodrug Users		Polydrug Users			
	*N *= 69,891		*n *= 35,875 (51.3%)		*n *= 34,016 (48.7%)		Odds Ratio	
				
Variable	*N*	%	*n*	%	*n*	%	OR^*a*^	95% CI
Gender								
Male	48,168	68.9	24,646	51.2	23,522	48.8	0.979^*c*^	0.948–1.011
Female	21,723	31.1	11,229	51.7	10,494	48.3	1.021^*c*^	0.989–1.055
Ethnicity								
Caucasian	46,910	69.2	26,218	55.9	20,692	44.1	1.957^*b*^	1.893–2.023
African American	20,393	30.1	7,941	38.9	12,452	61.1	0.504^*b*^	0.487–0.521
Other	495	0.7	269	54.3	226	45.7	1.154^*ns*^	0.967–1.379
Age								
<18	6,955	10.0	4,525	65.1	2,430	34.9	1.876^*b*^	1.782–1.976
18–24	10,701	15.3	5,111	47.8	5,590	52.2	0.845^*b*^	0.811–0.880
25–34	20,671	29.6	9,347	45.2	11,324	54.8	0.706^*b*^	0.683–0.730
35–44	21,893	31.3	10,833	49.5	11,060	50.5	0.898^*b*^	0.870–0.927
45–54	8,266	11.8	4,931	59.7	3,335	40.3	1.466^*b*^	1.399–1.536
≥ 55	1,404	2.0	1,127	80.3	277	19.7	3.950^*b*^	3.461–4.509
Residential Area								
Urban	35,062	50.2	17,035	48.6	18,027	51.4	0.802^*b*^	0.779–0.826
Rural	34,829	49.8	18,840	54.1	15,989	45.9	1.248^*b*^	1.210–1.285
Year								
1998	9,911	14.2	4,645	46.9	5,266	53.1	0.812^*b*^	0.778–0.847
1999	9,123	13.1	4,327	47.4	4,796	52.6	0.836^*b*^	0.800–0.873
2000	9,598	13.7	5,293	55.1	4,305	44.9	1.194^*b*^	1.144–1.247
2001	10,214	14.6	5,618	55.0	4,596	45.0	1.189^*b*^	1.139–1.240
2002	9,891	14.2	4,885	49.4	5,006	50.6	0.913^*b*^	0.875–0.953
2003	10,738	15.4	5,711	53.2	5,027	46.8	1.092^*b*^	1.048–1.138
2004	10,416	14.9	5,396	51.8	5,020	48.2	1.023^*ns*^	0.981–1.066

^*a*^Odds ratio of being a mono- versus a polydrug user in comparing a specific level of client demographics to all other demographic features. Odds in this table are calculated for the odds of being a *mono *vs. *polydrug abuser*, not for being male rather than female.

^*b*^*p *< 0.0001.

^*c*^*p *< 0.10.

^*ns*^Not significant.

Adolescents (those under 18 years of age) were almost twice as likely to be monodrug abusers (OR = 1.876, *p *< 0.0001). Adults from 18 to 44 years old were slightly more likely to be polydrug abusers (ORs < 0.90, *p*-values < 0.0001), while adults 45 and over were significantly more likely to be monodrug abusers, especially those 55 and older (OR = 1.466 for ages 45–54 and OR = 3.950 for ages ≥ 55). Admissions in urban settings were more likely to be polydrug abusers (OR = 0.802, *p *< 0.0001), whereas rural admissions were more likely to be monodrug abusers (OR = 1.248, *p *< 0.0001). Across the 7 study years, there was a small overall decline in polydrug abuse from 53.1% of admissions in 1998 to 48.2% in 2004, but the decline was not consistent, falling to a low of 44.9% in 2000, then rising to 50.6% in 2002 before resuming a general decline. There is no clear pattern across the 7 study years in terms of likelihood of mono- versus polydrug abuse. Other than in 1998 and 1999, when the likelihood of polydrug abuse was slightly higher, there was no difference in the likelihood of admissions for mono- or polydrug abuse from year to year.

## Discussion

This study revealed that for both mono- and polydrug abusers, alcohol was the predominant substance of choice, followed by cocaine and marijuana, findings consistent with those reported by other national surveys [2,17,20]. These three substances were also the most common in two- and three-substance combinations reported by this study sample–at least one of these substances was part of 13 of the top 14 substance pairings and at least two were found in 11 of the top 15 triads. This strongly concurs with earlier studies in which the most frequent polydrug combinations included alcohol, specifically marijuana with alcohol, followed by alcohol with other illicit drugs, and marijuana with other illicit drugs [2,9,10,17]. Whereas some other researchers used the general category of "illicit drugs," this study explicitly identified cocaine as the illicit substance. In terms of total overall abuse, cocaine abuse was second only to alcohol and was reported in the most frequently abused two- and three-substance combinations.

Despite these similarities, in many ways the results of this research are quite different from those reported in previous studies. We found slightly lower polydrug abuse rates than the national trends reported by the Treatment Episode Data Set (TEDS) [21]. In a report of longitudinal trends of substance abuse from 1994–2005, only 44.2% of all admissions in 2005 reported no substance abuse in addition to the primary substance (p.163). The TEDS data also indicated that roughly half of adolescents between the ages of 12 and 17 who were admitted for substance abuse treatment were being treated for a combination of both alcohol and marijuana, although that proportion has been steadily decreasing since 1999 to about 43% of admissions in 2005 (p.156). While the majority (55.1%) of admissions for treatment of alcohol did not report abuse of other substances, admissions with other substances as the primary substance of abuse reported roughly 24.7 to 41.7% being treated for a single substance (p.163). So far, TEDS has not reported polydrug data by state, preventing a direct comparison with the results of this research in Tennessee.

Examinations of demographic characteristics of polydrug abusers, such as age, gender, and ethnicity, in order to identify trends have found that, in general, polydrug abuse was lowest for those over 40 years old and highest among those under 20 [2,22-24]. For example, Chen and Kandel found a tendency for polydrug abusers to be young, with initiation of illicit polydrug abuse being extremely rare after age 20 [22]. The DHHS found that 65% of admissions under 20 reported polydrug abuse, whereas only 41% of admissions over 45 did so [2]. Examining polydrug abuse by age group among national admissions to publicly funded substance abuse treatment, Henderson found peak polydrug abuse among those younger than 40 [23]. Earleywine and Newcomb found that a majority of polydrug abuse took place among those age 28–32, who constituted 31% of the combination abusers of marijuana and alcohol, 28% of alcohol and illicit drugs other than marijuana, and 22% of marijuana with other illicit drugs [25]. Collins et al. found polydrug abuse in 29% of 12th graders studied [26]. The American Academy of Family Physicians reported the abuse of multiple drugs by more than two thirds of patients age 20–30 and among more than four fifths of those under 20 [27]. Several other studies found high levels of polydrug abuse among adolescents [17,23,28]. At the same time, research has suggested that the incidence of polydrug abuse may decline with age. For example, Raveis and Kandel determined that 85–95% of abusers, other than those who were marijuana and alcohol dependent, had ceased multiple drug abuse by age 28–29 [24], and Darke and Hall noted that among heroin and amphetamine abusers the number of drugs used tended to decrease with age [11]. In contrast to these previous studies, this research found that while polydrug abuse was higher among clients between the ages of 18 and 44, monodrug abuse prevailed among minors and adults over 55.

Earlier research also found that males exhibited more polydrug abuse than females [11,17,18]. In this study, however, males and females were equally likely to be polydrug abusers. With regard to ethnicity, our research differed from previous studies as well. Asians have been shown to be less likely than African Americans, Hispanics, or Caucasians to be polydrug abusers [15,18], and African Americans have been found to be less likely to be polydrug abusers than Hispanics or Caucasians [18]. Our findings indicated that Caucasians were more likely to be monodrug abusers and African Americans were more likely to be polydrug abusers, in direct contrast to prior reports of lower rates of polydrug abuse among African Americans than Caucasians [18]. Additionally, although there is little in the substance abuse literature regarding rural versus urban polydrug abuse in the United States, this study found that admissions in urban areas were more likely to be for polydrug abuse, while rural admissions were more likely to be for monodrug abuse.

The variation in study results discussed above suggests that accounting for regional differences in substance abuse patterns may be very important, if not determinant, in the implementation of public health policy at the state level. National estimates, even the state breakdowns provided in SAMHSA's National Survey on Drug Use and Health, may be less reliable than comprehensive, multiyear research derived from local treatment providers, such as the findings presented here.

## Conclusion

Although this study is based on a large dataset, it is limited in that the study sample consisted only of admissions to publicly funded treatment in Tennessee. In other areas of the country, there may be different patterns of mono- and polydrug abuse among those admitted to private treatment facilities, or among substance abusers in the general population not seeking treatment. The national data available from SAMHSA also comprised publicly funded admissions; this means that there is no other major dataset with which to compare publicly funded admissions with other types to discern differences, if applicable, between publicly funded and nonpublicly funded admissions. Finally, this research did not address several other variables that have been independently associated with polydrug abuse, including not being in treatment, using drugs intravenously, sharing injection paraphernalia, being an amphetamine abuser, and being partially or fully disabled and/or having co-occurring mental health problems [10,11,29]. Despite these limitations, this study highlights the prevalence of polydrug abuse, a phenomenon that is as common as monodrug abuse, and examines the probability of mono- versus polydrug abuse by demographic variables.

Most of what is known about substance abuse in the United States does not distinguish between mono- and polydrug abuse, a gap in the research that has potentially adverse implications for substance abuse prevention and treatment policies. The complex nature of mono- versus polydrug abuse and the frequency with which cases of primary abuse of one substance easily led to a pattern of polydrug abuse [13] indicates that substance abuse treatment professionals must have a full range of protocols available to help them provide optimal intervention services. Drug treatment interventions tailored to the specific characteristics of the population are generally more successful [30,31]. Protocols have already been developed specifically for adolescents [32,33], minorities [34], clients with co-occurring mental illness [35,36], and clients with HIV [34], to name a few. The present study provides valuable data about the prevalence and patterns of mono- versus polydrug abuse in a statewide treatment population, which can be used to assist in developing the most effective prevention and treatment plans for these populations.

Polydrug abuse has a significant public health impact. The interaction among multiple drugs can heighten the neurological, physiological, and psychological impact on the user as well as potentially increase the negative consequences of polydrug abuse. In addition, as Schensul et al. [37] notes " [c]umulative multiple drug use is associated with poorer physical health, greater likelihood of addiction, and other social and mental health problems" (p.571). For example, after examining data from the Drug Abuse Warning Network (DAWN), McCabe et al. determined that most emergency room visits related to drug intake involved polydrug abuse, especially the combination of prescription medication with alcohol [38]. The consequences may be even more serious; Cone et al. found that the overwhelming majority of oxycodone-related deaths between August 1999 and January 2002 involved another drug agent [39].

Areas for future investigation might address those specific aspects of polydrug abuse that might impact prevention and treatment. For example, what is the array of forces/stimuli motivating polydrug abuse, and how do these vary according to demographics? Is polydrug abuse chronic or simply one stage in the life of a substance abuser? If we followed a polydrug abuser across time, would we find periods when he or she was a monodrug abuser, and other times a polydrug abuser? The answers to these questions have important policy implications, especially in terms of providing polydrug abusers with the most effective treatment regimen.
